# Clinical and surgical outcomes of patients with peritoneal mesothelioma discussed at a monthly national multidisciplinary team video‐conference meeting

**DOI:** 10.1002/bjs5.50256

**Published:** 2020-01-30

**Authors:** A. Brandl, S. Westbrook, S. Nunn, E. Arbuthnot‐Smith, J. Mulsow, H. Youssef, N. Carr, A. Tzivanakis, S. Dayal, F. Mohamed, B.J. Moran, T. Cecil

**Affiliations:** ^1^ Peritoneal Malignancy Institute, Basingstoke and North Hampshire Hospital Basingstoke UK; ^2^ Good Hope Hospital, Heart of England NHS Foundation Trust Birmingham UK; ^3^ National Centre for Peritoneal Malignancy, Mater Misericordiae University Hospital Dublin Ireland

## Abstract

**Background:**

Peritoneal mesothelioma (PM) is a rare primary neoplasm of the peritoneum with an increasing incidence worldwide. Cytoreductive surgery (CRS) with hyperthermic intraperitoneal chemotherapy (HIPEC) has shown promise as a treatment strategy. A national PM multidisciplinary team (national PM MDT) video‐conference meeting was established in the UK and Ireland in March 2016, aiming to plan optimal treatment, record outcomes and provide evidence for the benefits of centralization. This article reports on the activities and outcomes of the first 2·5 years.

**Methods:**

Between March 2016 and December 2018, patients with PM, referred to peritoneal malignancy centres in Basingstoke, Birmingham, Manchester and Dublin, were discussed by the national PM MDT via video‐conference. The MDT was composed of surgeons, radiologists, specialist nurses and pathologists. Patients were considered for CRS and HIPEC if considered fit for surgery and if radiological imaging suggested that complete surgical cytoreduction could be achieved. Morbidity and mortality following surgery were analysed. Survival analysis following MDT discussion was conducted.

**Results:**

A total of 155 patients (M : F ratio 0·96) with a mean(s.d.) age of 57(17) years were discussed. To date, 22 (14·2 per cent) have had CRS and HIPEC; the median Peritoneal Cancer Index for the surgical group was 17·0. Complete cytoreduction was achieved in 19 patients. Clavien–Dindo grade I–II complications occurred in 16 patients; there was no grade III–IV morbidity or 30‐day in‐hospital mortality. The median follow‐up for the whole cohort was 18·7 months, and the 2‐year survival rate from time of first review at the national PM MDT was 68·3 per cent.

**Conclusion:**

The centralized national PM MDT was effective at selecting patients suitable for CRS and HIPEC, reporting a good outcome from patient selection.

## Graphical abstract

The focus of this study was to demonstrate experience in understanding, managing and treating peritoneal mesothelioma, a rare disease, and explore how this was facilitated through the establishment of a national multidisciplinary team (MDT), a model that may be useful for other rare diseases. This centralized national peritoneal mesothelioma MDT was effective at selecting patients suitable for cytoreductive surgery and hyperthermic intraperitoneal chemotherapy, and advising on the optimal treatment strategy for patients unlikely to benefit from surgery.

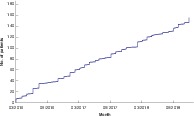

Peritoneal mesothelioma and national MDT

## Abstract (es)

**Antecedentes:**

El mesotelioma peritoneal (*peritoneal mesothelioma*, PM) es una neoplasia primaria del peritoneo muy poco frecuente, con una incidencia creciente en todo el mundo. La cirugía citorreductora (*cytoreductive surgery*, CRS) con quimioterapia intraperitoneal hipertérmica (*hyperthermic intraperitoneal chemotherapy*, HIPEC) se ha mostrado prometedora como estrategia de tratamiento. En marzo de 2016, se organizó una reunión por videoconferencia del equipo multidisciplinar nacional de PM (*national PM multi‐Disciplinary Team*, MDT) en el Reino Unido e Irlanda, con el objetivo de planificar un tratamiento óptimo, registrar los resultados y proporcionar evidencia de los beneficios de la centralización. Este manuscrito presenta las actividades y los resultados de los primeros 2,5 años.

**Métodos:**

Entre marzo de 2016 y diciembre de 2018, 155 pacientes con PM, remitidos a centros de cirugía oncológica peritoneal en Basingstoke, Good Hope Hospital en Birmingham, Christie Hospital en Manchester y Mater Misericordiae en Dublín, fueron discutidos en el National PM MDT a través de una videoconferencia. El MDT estaba compuesto por cirujanos, radiólogos, enfermeras especializadas y patólogos. Los pacientes fueron considerados para CRS e HIPEC si se determinaba que eran aptos para la cirugía y si las imágenes radiológicas sugerían que se podía lograr una citorreducción quirúrgica completa. Se analizó la morbilidad y mortalidad después de la cirugía. Se realizó un análisis de supervivencia tras la discusión en el MDT.

**Resultados:**

En total, se discutieron 155 pacientes (tasa varón/mujer 0,96) con una edad media de 57 ± 17 años. Hasta el momento, 22 (14,2%) habían sido sometidos a CRS y HIPEC y la mediana de PCI en el grupo quirúrgico fue de 17,0. La citorreducción completa se logró en 19 (86,4%), las complicaciones de Clavien‐Dindo grado I/II ocurrieron en 16/22, sin morbilidad de grado III/IV, ni mortalidad a los 30 días. La mediana de seguimiento fue de 15,0 meses y la supervivencia a los 2 años desde el momento de la revisión en el National PM MDT fue del 66,7%.

**Conclusión:**

El National PM MDT centralizado fue eficaz en la selección de pacientes adecuados para CRS e HIPEC, presentando un buen resultado a partir de dicha selección.

## Introduction

Mesothelioma is a rare malignancy originating from mesothelial cells of the serosal layers of the pleura, peritoneum, pericardium or tunica vaginalis testis. Peritoneal mesothelioma (PM) represents 7–10 per cent of mesothelioma diagnoses, and is the second most common site of origin after the pleura^1^. PM may present as a diffuse pattern with multiple tumour nodules throughout the abdominal cavity, or with isolated, localized masses. The epidemiology of PM is difficult to establish because of geographic and temporal variations in exposure to asbestos and challenges with disease diagnosis. In industrialized countries, the incidence is 0·5–3 cases per million in men and 0·2–2 cases per million in women^2^.

Diagnosis is often delayed due to presentation with non‐specific symptoms, and many patients present at an advanced tumour stage. Ascites and abdominal pain are common presenting symptoms, with weight loss, anorexia, a palpable abdominal mass or new‐onset hernia, due to ascites‐related increased abdominal pressure^1^, ^3^.

Histopathological diagnosis of PM may be challenging as tumour cells can mimic benign reactive lesions or simulate other metastatic neoplasms. Three main histological types of malignant PM have been described: epithelioid, sarcomatoid and biphasic^1^, ^4^, ^5^. Patients with biphasic and sarcomatoid subtypes have a significantly worse prognosis than those with the epithelioid subtype. Treatment options are limited, with poor outcomes^6^. In contrast, multicystic mesothelioma and well differentiated papillary mesothelioma are both low‐grade forms of PM with a more favourable prognosis. Thus, there are challenges and overlap in the radiological and histopathological differential diagnosis between malignant and more indolent PM, and with other peritoneal malignancies such as pseudomyxoma peritonei, ovarian tumours and colorectal peritoneal metastases.

No new systemic chemotherapeutic agents for the treatment of mesothelioma have been identified over the past decade, and optimal therapy remains a platinum agent and pemetrexed. A proportion of patients respond to chemotherapy, resulting in disease control rather than cure.

Cytoreductive surgery (CRS) and hyperthermic intraperitoneal chemotherapy (HIPEC) has been shown to be effective in peritoneal malignancy, particularly in patients with pseudomyxoma peritonei^7^. Evidence to support CRS and HIPEC for patients with PM is accumulating. Median overall survival for selected patients with PM treated by CRS and HIPEC ranges from 29·5 to 95 months^8^, ^9^, ^10^, ^11^.

Poor prognostic factors include age above 60 years, deep tissue invasion by tumour, residual disease after CRS, biphasic/sarcomatoid histology, metastatic lymph nodes, and mitotic count greater than 5 per 50 high‐power fields^12^. Recent studies^13^, ^14^ also suggest that overall survival after CRS and HIPEC may be influenced by the Ki‐67 proliferation index.

The first CRS with HIPEC in the UK and Ireland was performed at the Peritoneal Malignancy Institute (PMI), Basingstoke, in 1994; although used predominantly for appendiceal tumours, a small number of patients with PM were treated in subsequent years. In July 2015, NHS England concluded that there was insufficient evidence for the effectiveness of CRS and HIPEC in patients with PM, such that currently CRS and HIPEC are not funded routinely by the National Health Service, highlighting the challenges of obtaining evidence for the treatment of rare diseases.

A monthly national PM multidisciplinary team (national PM MDT) video‐conference meeting was established in the UK and Ireland (population approximately 70 million) in March 2016 to plan optimal treatment for patients, select patients likely to benefit from CRS and HIPEC, and record outcomes. The aim was to optimize patient outcomes, to provide evidence for the benefits of centralization by supporting patients and referring clinicians, to understand and quantify current practice and the magnitude of clinical burden of PM, and to gain support for national centralized funding of a PM service, similar to the existing pseudomyxoma peritonei services in the UK and Ireland.

This article reports the results of the national PM MDT 2·5 years after its implementation.

## Methods

### National peritoneal mesothelioma multidisciplinary team

The PMI Basingstoke initiated a monthly national PM MDT video‐conference incorporating North Hampshire Hospital in Basingstoke, the Christie Hospital in Manchester, Good Hope Hospital in Birmingham, and the Mater Hospital in Dublin. Core members included surgeons, radiologists, specialist nurses and pathologists. Oncologists with an interest in PM attend when possible. A dedicated PM nurse specialist was appointed, with funding support from the charity Mesothelioma UK, and PMI Basingstoke funds a dedicated PM MDT coordinator.

All patients with PM from UK and Ireland referred to any of the peritoneal malignancy units were discussed. Case reviews focused on clinical details, radiological imaging and review of histological findings. Discussions explored clinical presentation, symptoms, diagnostic pathways and histological confirmation, aiming to provide therapeutic recommendations. All centres could view radiological images and speak with, and visualize, all participants.

The core components and infrastructure of the virtual MDT were a video system (web‐based telephone system) requiring multisite coordination with available technology at all sites. All centres sent confidential e‐mails for referrals to the lead MDT clinician. The coordinator of the national PM MDT gathered and checked all clinical information, prepared the case for presentation, and documented the data/outcome. Letters were dictated during the meeting and sent to the referring team. A key role was taken by the specialist nurse, who acted as key worker and patient advocate at the MDT, advised on suitable clinical trials, signposted support for patients, and referred them to other services.

The first national PM MDT was held in March 2016. Centres agreed that surgical treatment would be performed at one dedicated centre (PMI Basingstoke) for UK patients in order to provide consistent data on operative outcomes and cost. Patients residing in the Republic of Ireland could undergo treatment in Dublin.

Every patient referred to one of four nationwide dedicated peritoneal malignancy centres with a diagnosis of malignant mesothelioma, multicystic mesothelioma or well differentiated papillary mesothelioma was presented and discussed at the national PM MDT.

### Clinical data, survival and follow‐up

Several patient‐related factors were evaluated before the national PM MDT discussion: age, exposure to asbestos, clinical symptoms, previous chemotherapy, drug combinations, tumour response, previous surgical treatment, preoperative histology and baseline tumour markers (carbohydrate antigen (CA) 125, carcinoembryonic antigen, CA19‐9). Follow‐up for all surgically treated patients was performed predominantly via telephone interview by specialist nurses, and some by clinical consultations. In addition, follow‐up for all patients was completed by a request via the Demographics Batch Service to the NHS Spine, Personal Demographics Service, on 10 May 2019.

Patients who died before or on the day of the national PM MDT were excluded from survival analysis. Survival was calculated from date of presentation at the national PM MDT to last follow‐up. Details of postoperative complications were categorized according to the Clavien–Dindo classification^15^. Survival analysis was performed in patients with histologically proven mesothelioma.

### Histopathological data

Referring hospitals were asked to send the slides from relevant specimens for review by pathologists at Basingstoke with a special interest in PM. Tissue blocks were also requested if required for additional staining or immunohistochemistry. PM was classified into established histological subtypes^16^.

### Selection for surgery

Patients were considered for CRS and HIPEC if considered fit for surgery and if radiological imaging suggested that a CC0 (no visible disease) or CC1 (nodules smaller than 2·5 mm) surgical cytoreduction could be achieved. Extensive small bowel involvement, without the possibility of complete surgical removal whilst maintaining adequate remaining intestine, was the main factor making this unlikely. Other factors considered were the extent of disease, time from diagnosis, response to systemic chemotherapy, pathology and the Ki‐67 proliferation index. Staging laparoscopy was used to clarify the extent of disease and small bowel involvement. Recommendations for treatment were based on the clinical details, performance status, histological analysis and radiological assessment. The Peritoneal Cancer Index (PCI) and Ki‐67 were taken into consideration if the Ki‐67 proliferation index was above 9 per cent and the PCI was greater than 17, although cut‐off values were not applied strictly^13^.

Owing to the pattern of spread and advanced disease at diagnosis, almost all patients required a complete parietal peritonectomy including the peritoneum of the left and right diaphragm, pelvic peritonectomy, peritonectomy of the small bowel mesentery and paracolic gutters, together with greater and lesser omentectomy as originally described by Sugarbaker^12^. In addition, multivisceral resections were commonly required to achieve a complete cytoreduction. The drug combinations of cisplatin with doxorubicin or mitomycin C are used most commonly for HIPEC in patients with PM.

### Statistical analysis

All statistical analyses were performed using either SPSS® 23.0 (IBM, Armonk, New York, USA) or Prism® 6.0 (GraphPad, La Jolla, California, USA). Continuous descriptive data are given as mean(s.d.) or median (range) values. Categorical data are given as frequencies. Univariable survival analysis was conducted by Kaplan–Meier analysis. *P* < 0·050 was considered statistically significant.

## Results

**Figure 1:**
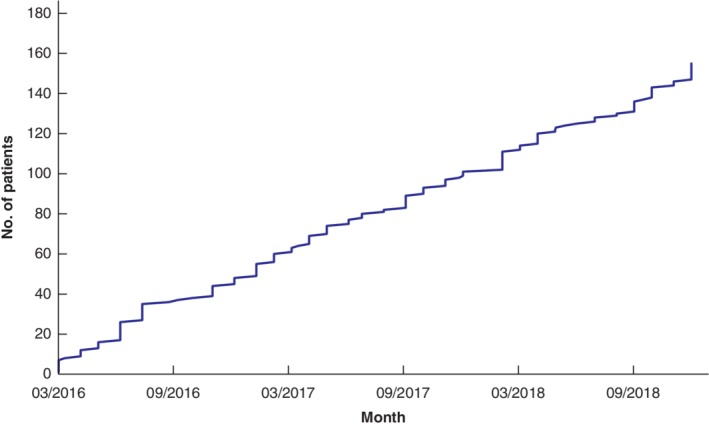
Total number of new patients discussed by the national peritoneal mesothelioma multidisciplinary team over time

In total, 34 national PM MDT meetings incorporated review of 155 new patients with PM (79 women and 76 men; mean age 57(17)) between March 2016 and December 2018. Overall, 35 patients were discussed more than once. The total number of new patients discussed at the national PM MDT over time is shown in *Fig*. 1.

Surgical outpatient assessment was recommended for 40 patients (25·8 per cent) and diagnostic laparoscopy in 12 patients (7·7 per cent).

**Figure 2:**
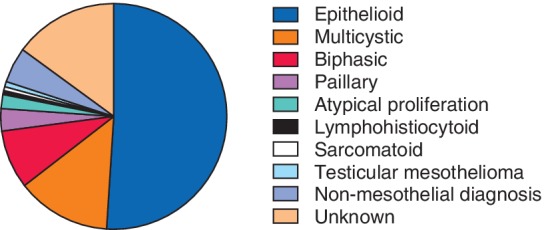
Pie chart showing fractions of different histopathological subtypes of peritoneal mesothelioma discussed by the national peritoneal mesothelioma multidisciplinary team

Histopathological subtype was available for 132 patients at the time of the national PM MDT review, with the epithelioid subtype in 79, sarcomatoid in one and biphasic in 13. Multicystic mesothelioma was the diagnosis in 21, papillary in five and lymphohistiocytoid in one patient. Three patients had atypical mesothelial proliferation with suspicious clinical and radiological presentation and one patient had testicular mesothelioma (*Fig*. 2).

In total, eight cases referred to the national PM MDT were eventually recategorized as not having a diagnosis of PM. Of these, two patients had a known different cancer and a second pathology opinion was being requested from the national PM MDT pathologist and four had a known other cancer opportunistically discussed to obtain a second opinion from other peritoneal malignancy surgeons. The remaining two patients were considered to have PM by the national PM MDT but the final diagnosis was changed following definitive pathology after CRS and HIPEC in one patient and diagnostic laparoscopy in one patient. In 23 cases, histological data were not sent for review, so that subtyping was not possible.

### Surgical treatment

All 22 patients (14·2 per cent of the 155 patients) undergoing CRS and HIPEC were treated in Basingstoke. The median PCI was 17 (range 6–39) and the mean(s.d.) duration of surgery was 405(116) min, with mean blood loss of 820(467) ml (*Table* 1). The mean ICU and postoperative hospital stay were 1·4(0·7) and 18·5(6·6) days respectively. Complete cytoreduction was achieved in 19 of the 22 patients. Clavien–Dindo grade I–II complications occurred in 16 patients; there was no grade III–IV morbidity. There were no postoperative deaths or reoperations.

**Table 1:** Demographics and operative data for patients with peritoneal mesothelioma treated with cytoreductive surgery and hyperthermic intraperitoneal chemotherapy

	CRS and HIPEC (*n* = 22)
**Age (years)** *	49·5(18·4)
**Sex ratio (F** : **M)**	14 : 8
**Duration of surgery (min)** *	405(116)
**Blood loss (ml)** *	820(467)
**PCI** †	17 (6–39)
**Complete cytoreduction**	19
**Histopathology**	
Cystic	4
LAMN	1
Endometriosis	1
Epithelioid	13
Biphasic	2
Atypical mesothelial proliferation	1
**Ki‐67 proliferation index (%)**	
≤ 9	3
> 9	6
**ICU stay (days)** *	1·4(0·7)
**Postoperative hospital stay (days)** *	18·5(6·6)
**Postoperative complications** ‡	
Surgical complications‡	
Bleeding	0
Pancreatic fistula	1
Prolonged ileus (> 14 days)	1
Pneumothorax	1
Wound infection	2
Reoperation	0
Medical complications‡	
Renal impairment	0
Pneumonia	1
Pulmonary embolism	2
Urinary tract infection	4
Neurological (hallucination)	10
Clavien–Dindo grade	
0	6
I–II	16
III–IV	0
V (death)	0
**Died during follow‐up**	4

Values are

* mean(s.d.) and

† median (range).

‡ Multiple parameters per patient possible. CRS, cytoreductive surgery; HIPEC, hyperthermic intraperitoneal chemotherapy; PCI, Peritoneal Cancer Index; LAMN, low‐grade appendiceal mucinous neoplasm.

Histological analysis following treatment with CRS and HIPEC showed that 19 patients had histologically proven PM. Of these, 13 had epithelioid, two had biphasic and four had multicystic PM subtypes. Non‐mesothelioma histology was identified in three patients who had CRS and HIPEC (low‐grade mucinous carcinoma peritonei of the appendix, 1; endometriosis, 1; atypical mesothelial proliferation, 1) (*Table* 1).

### Patient survival

Median follow‐up was 15·0 months. During this time, 43 of the 155 patients (27·7 per cent) died.

**Figure 3:**
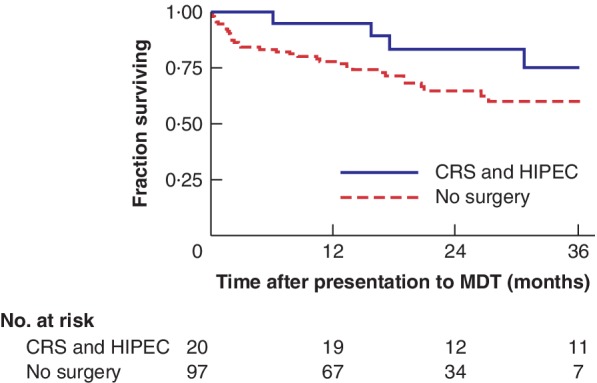
Kaplan–Meier analysis comparing overall survival in patients with peritoneal mesothelioma treated with cytoreductive surgery and hyperthermic intraperitoneal chemotherapy and patients managed without surgery CRS, cytoreductive surgery; HIPEC, hyperthermic intraperitoneal chemotherapy; MDT, multidisciplinary team. *P* = 0·138 (log rank test).

A total of 149 patients were included for survival analysis, as six died before or on the day of the national PM MDT. Overall, 43 patients died during the observation period, with 1‐, 2‐ and 3‐year survival rates of 81·0, 68·3 and 62·6 per cent respectively. Median overall survival was not reached. Patients selected for CRS and HIPEC had better 1‐, 2‐ and 3‐year overall survival rates (95·0, 83·2 and 75·1 per cent respectively) than those treated with systemic chemotherapy (78·1, 64·8 and 60·1 per cent), but the difference was not significant (*P* = 0·138) and may represent the short follow‐up and small numbers of patients (*Fig*. 3).

## Discussion

The incidence, prevalence and epidemiology of PM remain unclear. It is a rare disease and much less common than pleural mesothelioma. Pleural mesothelioma has been studied extensively, and management and outcomes reported, since links with asbestos exposure were first proposed in 1924^17^. From available data, the incidence of PM in the UK is estimated to be around 0·5 cases per million population^18^. The National Mesothelioma Audit Report^19^ documented that 4 per cent of mesothelioma cases diagnosed in England were PM (260 of 7192 cases in 2014–2016). The National Cancer Registry of Ireland listed an incidence of PM of 1·5 cases per million in men and 0·2 cases per million in women. In total, 41 patients were diagnosed with mesothelioma in 2014, with an estimate of three cases of PM in the same period^20^. Extrapolation from these data suggests an annual incidence of PM in the UK and Ireland of approximately 90 patients. During the observation period of 34 months, there were 255 patients diagnosed with PM in the UK and Ireland according to this model, some 155 of whom (60·8 per cent) were discussed by the national PM MDT.

Systemic chemotherapy for mesothelioma has failed to deliver the remarkable improvements in survival seen for other tumour types over the past two decades. For most patients with PM, treatment options are limited and the experience of the national PM MDT suggests that many patients present at an advanced stage. The role of CRS and HIPEC remains controversial, although in reality only a small proportion of patients were deemed suitable for this treatment by the national PM MDT. In total, 206 reviews of 155 patients were undertaken over a 34‐month interval, with only 22 patients (14·2 per cent) deemed suitable for surgery. The remaining patients had either palliative systemic chemotherapy or best supportive care.

Prognostic indicators may play a role in identifying those likely to benefit from surgery, but a number of these indicators are available only after laparotomy and CRS when histological information such as tumour type, lymph node involvement and Ki‐67 index are assessed. The present results suggest that discussing patients at a national PM MDT helps to select patients most likely to benefit from intervention. Radiology is crucial, and an experienced peritoneal malignancy radiologist helps to identify unfavourable anatomical sites of disease that preclude complete cytoreduction such as extensive small bowel serosal and/or porta hepatis disease^21^. CT is currently the main imaging modality, but there may be a role for diffusion‐weighted MRI, particularly when assessing small bowel involvement^22^.

The national PM MDT also highlighted the challenges in making an accurate histological diagnosis in patients with PM. Of the 22 patients referred by the national PM MDT for CRS and HIPEC, three nevertheless had a discordant non‐mesothelioma diagnosis on histological analysis compared with the preoperative diagnosis and national PM MDT decision. It is of vital importance that an experienced pathologist has the opportunity to review histology slides in such a rare malignancy, of which most pathologists may have limited experience.

The largest published multicentre retrospective study^23^ of patients with PM included 405 patients from 29 centres in the USA and Europe, and reported a median survival of 53 months and a 5‐year overall survival rate of 47 per cent after CRS and HIPEC.

Survival following systemic chemotherapy with pemetrexed and cisplatin is poor. Based on US national data from over 1000 patients with pleural and peritoneal mesothelioma^24^, the overall response rate was 26 per cent and the stable disease rate 45 per cent, with a combined disease control rate of 71 per cent. The treatment was generally well tolerated; reported severe adverse events were primarily non‐haematological and included dehydration (7·2 per cent), nausea (5·2 per cent) and vomiting (4·9 per cent). Overall survival of patients treated with chemotherapy alone was approximately 13 months, suggesting that the duration of clinical benefit is short.

A previous study^25^ analysed the US national cancer database, which included a range of management options such as best supportive care, systemic chemotherapy, CRS, and CRS with HIPEC, in 1514 patients with PM. Patients treated with CRS or CRS and HIPEC with systemic chemotherapy, had longer overall median survival than those who had systemic chemotherapy alone, or best supportive care (52 months after CRS, 61 months after CRS and HIPEC, 17 months after systemic chemotherapy, and 6 months for best supportive care; *P* < 0·001).

In a study^26^ of the Finnish national experience with PM in 2000–2012, a total of 90 patients were diagnosed with PM over a 12‐year period, representing an incidence of 0·74 new cases per million, per year, in Finland. Surgical intervention was performed in 14 of 90 patients (16 per cent) in two different centres, while 37 patients (41 per cent) were treated with systemic chemotherapy and 14 (16 per cent) had radiotherapy. The median overall survival in patients who had complete cytoreduction was 59 months. Of note, 52 of the 90 patients (58 per cent) had lymph node spread beyond the regional lymph nodes at the time of diagnosis in a disease that is traditionally thought to remain confined to the peritoneal cavity.

Recent evidence^27^ suggests that new treatment modalities such as pressurized intraperitoneal aerosol chemotherapy (PIPAC) may have a role in achieving locoregional disease control. Theoretically, PIPAC combined with systemic chemotherapy might be an effective option for patients in whom CRS and HIPEC is not achievable. A retrospective single‐centre study^28^ demonstrated safe administration of PIPAC and histological regression in the majority of 29 patients treated with doxorubicin and cisplatin. Establishing a streamlined treatment pathway, integrating a national MDT and centralizing expertise, will allow optimization of care for patients with this disease and help to initiate clinical trials to identify the most effective treatment strategies in the future.

Many patients present with PM at an advanced stage and are not suitable for CRS and HIPEC. Systemic chemotherapy is of limited benefit, but is the only treatment option available for the majority of patients. Combined treatment algorithms (intraperitoneal and systemic) may optimize tumour control in patients unsuitable for CRS and HIPEC, and further evaluation is required.

A monthly national PM MDT video‐conference provides cost‐effective optimal therapeutic strategies and allows case selection for expensive, highly effective, treatment incorporating CRS and HIPEC. Good outcomes can be achieved in carefully selected patients through a national MDT process. A similar strategy of a national MDT by video‐conference may have applications in many other rare diseases and warrants ongoing evaluation. This service evaluation provides evidence that a national PM MDT makes important contributions to the management of patients with PM.

## Acknowledgements

S.W. is supported by a charitable grant from Mesothelioma UK.

*Disclosure*: The authors declare no conflict of interest.
